# Establishing a critical care echocardiography laboratory

**DOI:** 10.1186/cc12122

**Published:** 2013-03-19

**Authors:** K Yastrebov, A McLean

**Affiliations:** 1 The St George Hospital, Sydney, Australia; 2 Nepean Hospital, Sydney, Australia

## Introduction

Echocardiography is increasingly utilized by intensive care physicians in everyday practice. Standardization of echocardiographic studies and reporting, quality assurance and medicolegal requirements necessitate establishment of a dedicated system within the critical care setting. We describe the process of setting up a critical care echocardiography (CCE) laboratory based on our experience from three separate ICUs.

## Methods

A retrospective review and analysis of the process involved in establishment of echocardiography laboratories within ICUs.

## Results

Creating a CCE service involves a number of stages and takes several years to achieve. Major components include staffing, equipment, quality control, study archiving and networking capability. For staffing the objective is to identify and recruit staff with adequate training and expertise in CCE, providing 24/7 specialist cover in addition to supporting and training junior medical and nursing staff. There is further a need to acquire funding for high-quality ultrasound machines and related hardware as well as long-term DICOM-based archiving and reporting systems. This should be based on projections of annual volumes of echo studies and corresponding digital storage. Networking connectivity is highly desirable, including obligatory back-up solutions and site allocations. A business case incorporating all the above should precede any development as identifiable funding sources and administrative approval are essential. The implementation stage requires the presence of a project leader who can organize the trialing of scanners, archiving, reporting and research systems, ensure compatibility with existing hospital and cardiology networks, and who can assist in individualizing archiving and reporting software reflecting institutional and ICU specifics. Coordination with the IT department is very important. Clear contractual vendor obligations for service, maintenance and future upgrades of hardware and software need to be specified. Training and credentialing of staff is best achieved within a systematic framework that includes ongoing competency review, education and QA programs. Partnership with cardiology may benefit both groups. Major pitfalls are associated with poor initial training, lack of expertise and leadership, and bad vendor contracts.

## Conclusion

Establishment of a CCE laboratory requires careful planning, and allocation of adequate human and financial resources. Many potential problems can be identified and prevented in advance. Strong expert leadership plays an important role.

